# Improving Predictions of COVID-19 Preventive Behavior: Development of a Sequential Mediation Model

**DOI:** 10.2196/23218

**Published:** 2021-03-17

**Authors:** James A Roberts, Meredith E David

**Affiliations:** 1 Marketing Department Hankamer School of Business Baylor University Waco, TX United States

**Keywords:** pandemic, COVID-19, preventive behavior, self-efficacy, prevention, behavior, modeling, student, communication

## Abstract

**Background:**

Since the beginning of the COVID-19 pandemic, social distancing, self-quarantining, wearing masks, and washing hands have become part of the new norm for many, but not all. It appears that such preventive measures are critical to “flattening the curve” of the spread of COVID-19. The public’s adoption of such behaviors is an essential component in the battle against what has been referred to as the “invisible enemy.”

**Objective:**

The primary objective of this study was to develop a model for predicting COVID-19 preventive behaviors among US college students. The Health Belief Model has a long history of use and empirical support in predicting preventive health behaviors, but it is not without its purported shortcomings. This study identifies a more optimal and defensible combination of variables to explain preventive behaviors among college students. This segment of the US population is critical in helping slow the spread of COVID-19 because of the relative reluctance of college students to perform the needed behaviors given they do not feel susceptible to or fearful of COVID-19.

**Methods:**

For this study, 415 US college students were surveyed via Qualtrics and asked to answer questions regarding their fear of COVID-19, information receptivity (seeking relevant information), perceived knowledge of the disease, self-efficacy, and performance of preventive behaviors. The PROCESS macro (Model 6) was used to test our conceptual model, including predictions involving sequential mediation.

**Results:**

Sequential mediation results show that fear of COVID-19 leads individuals to seek out information regarding the disease, which increases their perceived knowledge and fosters self-efficacy; this is key to driving preventive behaviors.

**Conclusions:**

Self-imposed preventive measures can drastically impact the rate of infection among populations. Based on this study’s newly created sequential mediation model, communication strategies for encouraging COVID-19 preventive behaviors are offered. It is clear that college students, and very possibly adults of all ages, must have a healthy fear of COVID-19 to set in motion a process where concerned individuals seek out COVID-19–related information, increasing their store of knowledge concerning the disease, their self-efficacy, and ultimately their likelihood of performing the needed preventive behaviors.

## Introduction

The COVID-19 pandemic is the most recent and devastating of several viral outbreaks that occurred in the past 10-12 years, which include the H1N1 (“swine flu”), severe acute respiratory syndrome (SARS), and Middle East respiratory syndrome (MERS) outbreaks. As of December 10, 2020, there have been 15,040,175 COVID-19 cases diagnosed in the United States and 69,598,919 confirmed cases worldwide. As a result of the COVID-19 pandemic, 285,351 people have died in the United States and 1,582,665 people have died worldwide [1,2]. Social distancing, self-quarantining, wearing masks, and washing hands have become part of the new norm for many, but not all.

It appears that such preventive measures are critical to “flattening the curve” of the spread of COVID-19. The public’s response to prevention messages is an essential component in the battle against what has been referred to as the “invisible enemy” [3]. Effective marketing (preventive) messages that encourage people to social distance, wash their hands, wear masks, and avoid crowds will be critical to ending (or slowing) the spread of COVID-19. However, previous research has shown that increasing awareness of health-related topics is not the same as getting individuals to perform the preventive behaviors needed to avoid being infected and further spreading disease [4].

### The Health Belief Model

The Health Belief Model (HBM) had its beginnings in the 1950s. The initial version of the HBM was created by Godfrey Hocbaum, Stephen Kegels, and Irwin Rosenstock in 1952 [5]. At the time, Hocbaum, Kegels, and Rosenstock were working for the US Public Health Service. The original purpose of the HBM was to better understand why people did not partake in public health services such as chest X-rays to screen for tuberculosis. The model was an attempt to integrate Stimulus Response Theory [6] with Social Cognitive Theory (SCT) [7] to better understand various health-related behaviors. Many authors have noted the similarities between the HBM and SCT. SCT, similar to the HBM, is based upon Expectancy-Value Theory (EVT) [8].

SCT stresses the importance of an individual’s subjective valuations and his or her expectation that a certain behavior (eg, social distancing during the current pandemic) will lead to the desired end (avoidance of the COVID-19 virus). This combined approach to encouraging healthy behavior is grounded in EVT, based on the early work of Lewin [9] and Vroom [10] and later work by Fishbein and Ajzen [11] and Eccles and several coauthors [8,12-14]. Lewin’s theories espoused that it is perceptions of reality, rather than reality itself, that impact behavior [15]. The linchpin of EVT is that incentives or reinforcers do not directly impact health-related behavior but do so by impacting an individual’s assessment of the behavior requested and the expectation that said behavior will produce the desired results [8].

The original HBM included four variables: perceived susceptibility (likelihood of contracting the disease), perceived severity (the seriousness/danger of catching the disease), perceived barriers (factors that make taking the required actions difficult), and perceived benefits (positive results of enacting the needed behaviors). In 1988, Rosenstock et al [8] extended the HBM by including a measure of self-efficacy (ie, a person’s perception of his or her ability to perform the needed health-related actions). Cues to actions, such as perceived COVID-19 symptoms, personal advice, exposure to media messages, or any other factors that prompt action, are also often included in current applications of the HBM. Over the years, the HBM has been used to explain a wide variety of preventive health behaviors, including during other viral outbreaks (eg, H1N1, SARS, and MERS) similar to the current COVID-19 outbreak [16-18].

Despite the HBM’s long history of use and empirical support [8,16,19], it is not without purported shortcomings [20,21]. Several research studies suggest that the percentage of variance explained by the HBM across a variety of preventive measures is low (average *R*^2^ of about 21%) [21] and that the original HBM and its extensions have not used an optimal combination of its most commonly used predictor variables.

Manika and Golden [16] tested an extended version of the HBM. The authors examined the predictive ability of five variables in explaining the likelihood of engaging in preventive behaviors related to the H1N1 pandemic: perceived knowledge, stored knowledge, perceived threat, perceived self-efficacy, and information receptivity (seeking). The researchers entered all five variables simultaneously into a regression model. Of the five independent variables entered into the equation, information receptivity and perceived threat had the strongest coefficients (.39 and .35, respectively). The overall model’s *R*^2^ was .46. Later researchers noted that this may not be an optimal combination of predictor variables [20,21]. All the predictor variables were entered into the regression analysis simultaneously.

Orji et al [21] added four new variables to Manika and Golden’s [16] extension of the HBM: self-identity, perceived importance, consideration of future consequences, and concern for appearance. The authors also tested all possible relationships/interactions between the original HBM variables. In testing the ability of their extended HBM to predict healthy eating habits, the authors found that the extended model had an *R*^2^ of .71 compared to .40 for the original HBM. One’s belief that one can do what is needed to enact behaviors (self-efficacy) was found to be the strongest predictor of preventive behaviors. Results also shed light on several possible combinations of the original HBM variables that could help better explain individual health-seeking behavior.

Jones et al [20] also found that alternative combinations of the HBM’s variables can improve its predictive ability. The authors conducted a survey of 1337 Indiana residents after an 8-month flu vaccine campaign that was based on the HBM. Initial findings showed that exposure to the campaign increased the likelihood of getting a flu shot. The study also found an indirect effect of exposure on vaccine behavior through perceived barriers and that perceived threat was moderated by self-efficacy. Perceived barriers and benefits were also part of a serial mediation chain. The authors conclude that the relationships between the variables of the HBM are complex, may help explain earlier inconsistent results, and should be an important focus of future research.

### An Improved Model for Predicting COVID-19 Preventive Behavior

**Figure 1:**
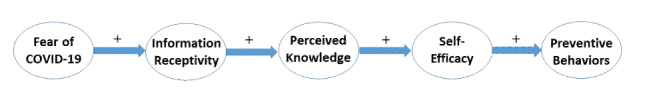
Conceptual model.

The present model (Figure 1) extends the additive model tested by Manika and Golden [16]. Extensions include the following: (1) use of the newly created and validated “fear of COVID-19” measure [22], (2) use of three separate measures of health-seeking behaviors (including measures developed by Masuri et al [17] and Manika and Golden [16], as well as a brief measure of social distancing created for this study) as the study’s dependent variables, and (3) a new combination of predictor variables. We identify and test a sequential mediation model that uses four of the five predictor variables used in the Manika and Golden model. The current model does not include stored knowledge as an independent variable because the Manika and Golden study [16] found that it is not actual but perceived knowledge that impacts preventive health behaviors.

Fear of COVID-19 [22] begins the process of encouraging COVID-19 preventive behaviors. As stated by Ahorsu et al [22], “One characteristic nature of infectious disease compared with other conditions is fear.” The scale was developed specifically to gauge fear of COVID-19 and was found to be a valid measure. If an individual thinks that they are not susceptible to getting the virus and that the virus is not a serious threat, they are less likely to engage in preventive behaviors [16,20]. A good example is the 2020 college spring break in the United States, where many students congregated on the nation’s beaches during the COVID-19 pandemic because they did not feel that COVID-19 was a serious threat [23].

If someone is fearful of the COVID-19 virus, we hypothesize that they are more likely to search for information that can help them avoid contracting the disease. This is the information receptivity variable in the Manika and Golden [16] version of the HBM. Moorman and Matulich [24] tested a similar model to explain preventive health behaviors and found that individuals with higher levels of health motivation were more likely to search for health-related information than less motivated individuals. The authors define health motivation as the arousal level to engage in preventive health behaviors. Items of their health motivation scale measured anxiety or worry about risks to one’s health. Hence, fear of COVID-19 will likely increase one’s motivation to search for COVID-19–related information.

This increased search for information regarding the COVID-19 virus is hypothesized to lead to a greater level of perceived knowledge. With the emergence of the COVID-19 pandemic, there has been a flood of both information and misinformation about the virus [25]. Depending on the person and media accessed, this increased access to information may or may not lead to appropriate health-related COVID-19 preventive behaviors. Manika and Golden [16] found that perceived knowledge quantity regarding the H1N1 pandemic was positively associated with H1N1 prevention behaviors.

Higher levels of perceived knowledge are hypothesized to lead to greater self-efficacy. Self-efficacy is best understood as an individual’s perception that he or she can perform the suggested health-related behaviors. The more confident an individual is that he or she can perform a certain task, the more likely it is that they will engage in the relevant preventive behavior [26]. In Moorman and Matulich’s [24] comprehensive review of the preventive health behavior literature, “health ability,” a person’s perceived ability to perform health behaviors, was found to predict preventive health behaviors. Self-efficacy has been found to be the most important predictor of health-related behaviors [4,21]. Being equipped with perceived knowledge regarding the behaviors needed to avoid contracting COVID-19 and having the confidence that one can perform these behaviors will increase the likelihood of performing COVID-19 preventive behaviors.

To summarize, the sequential mediation model presented and tested in this study hypothesizes that COVID-19 preventive behaviors are driven by a process of sequential mediation in which fear of COVID-19 leads to information receptivity, which in turn leads to perceived knowledge that drives self-efficacy and ultimately results in preventive actions. This proposed sequential mediation model is a potentially significant improvement over previous attempts to predict a variety of health-related behaviors using the HBM or variations thereof. The frequent use of an “additive model” approach to entering independent variables has come under scrutiny and calls have been made to explore other possible causal models [19,27]. This call has been echoed by Champion and Skinner [28], who see the need to better define the relationships between the predictor variables of health-seeking behavior. Jones et al [20] state that establishing a more optimal ordering of such variables will “advance theory and practice by improving evaluation, identifying relative importance of the constructs, and suggesting new postulates for behavior change.”

This study’s results provide such insights and offer clear directions on what types of preventive messages will be most effective in motivating individuals to incorporate behaviors into their daily routine that help keep them safe from COVID-19. Given the severity of the consequences (both personal and economic) of the COVID-19 pandemic and the importance of individual behaviors in “flattening the curve,” research that expands our understanding of what needs to be done to combat this “invisible enemy” is critical.

## Methods

### Overview

The study design included a cross-sectional survey designed in Qualtrics, which was administered online to participants, who completed the study in one sitting at a time that was convenient for them. The study data were collected using Qualtrics to administer questionnaires to 415 undergraduate students in the United States (49% male; mean age 21 [SD 5.41] years). Participants were recruited based on being enrolled in a subject pool at the authors’ university. Data inclusion criteria were broad such that the subject population included students across multiple majors and class identifications who were aged ≥18 years and participated in the study in exchange for course credit. There were no exclusion criteria and responses were accepted from all students who chose to complete the study. In an effort to minimize any potential response bias, participants were provided with a consent form to complete as part of the study, in which they were informed that their participation is voluntary, they can choose to terminate their participation in the study at any point if they so desire, and they will not be penalized if they choose not to participate in the study.

In addition, informed consent forms were completed by all study participants, in which they were informed that there were no risks involved in participating in the study and that their responses and all data collected would be recorded and entered into a spreadsheet for analysis in a manner whereby all participants will remain anonymous to the researchers, no personal identifying information would be requested, and all data would remain confidential. Most participants were White (n=313, 75%), followed by Hispanic (n=47, 11%), Asian (n=32, 8%), and African American (n=15, 4%).

### Measures

#### Fear of COVID-19

Fear of COVID-19 (*α*=.89, mean 2.06 [SD 1.87]) was assessed using the 7-item fear of coronavirus-19 scale developed and validated by Ahorsu et al [22]. Example items include “It makes me uncomfortable to think about coronavirus-19,” “When watching news and stories about coronavirus-19 on social media, I become nervous or anxious,” and “My hands become clammy when I think about coronavirus-19.” Response categories range from “strongly disagree” (1) to “strongly agree” (5).

#### Information Receptivity

Information receptivity (*α*=.84, mean 4.02 [SD 1.53]) was measured using items adapted from Manika and Golden’s [16] 3-item Information Receptivity Scale, which assessed how receptive individuals were to H1N1 information and how actively they sought such information. An example item is “I actively search for information about the H1N1 (swine) flu,” which was adapted in this study to “I actively search for information about the coronavirus (COVID-19).” Participants indicated their agreement with the three items using a 7-point scale anchored with strongly disagree and strongly agree.

#### Perceived Knowledge

Participants’ perceived knowledge (*α*=.84, mean 3.54 [SD .80]) was assessed using an established 4-item measure [16]. Specifically, participants used a 5-point response format (where 1=nothing and 5=a lot) to respond to the following statements: “In general, how much do you think you know about COVID-19,” “How much do you think you know about protecting yourself from getting COVID-19,” “How much do you think you know about the ways a person can and cannot get COVID-19,” and “Please rate your knowledge of COVID-19 compared to the average person.”

#### Perceived Self-Efficacy

Perceived self-efficacy (*α*=.91, mean 5.56 [SD 1.11]) was assessed using 5 items adapted from the Manika and Golden [16] scale, which was based on Bandura’s [7] concept of self-efficacy and assessed how confident individuals felt in their ability to make H1N1 flu prevention choices. An example item is “How confident do you feel about your ability to use your knowledge of H1N1 (swine) flu in making everyday activity choices,” which was adapted in this study to “How confident do you feel about your ability to use your knowledge of COVID-19 in making everyday activity choices?” Participants responded to the items on a 7-point scale anchored with not at all confident and completely confident.

#### Prevention Behavior

The Prevention Behavior Scale by Manika and Golden [16] was used to measure prevention behaviors (*α*=.85, mean 6.90 [SD 2.17]). The original scale includes the following 4 items: “It is important to me to do everything I reasonably can to avoid getting the H1N1 (swine) flu,” “I actively seek information on how I can prevent myself from getting the H1N1 (swine) flu,” “I am doing all that I know to do to prevent myself from getting the H1N1 (swine) flu,” and “I have changed my behavior to try to avoid getting the H1N1 (swine) flu.” For this study, “H1N1 (swine) flu” was changed to “COVID-19.” Participants responded to the items using a 7-point Likert scale anchored with strongly disagree and strongly agree. We included two additional measures of prevention behavior that were aimed at more directly assessing changes in behaviors related to COVID-19 prevention. Specifically, we included a measure of actual changes in behaviors, measured using the Masuri et al [17] scale, which is a list of 10 health-related behaviors that participants answer with a “yes” or “no” response. In addition, we included two items that made up the social distancing measure developed for this study (*α*=.92). These additional measures are reflective of the primary thrust of current preventive message campaigns to social distance to protect oneself and to avoid spreading COVID-19.

### Data Analysis

The study data were analyzed in SPSS Statistics (version 25; IBM Corp). Since data for all the study measures were obtained from the same source, common method bias could be a potential issue. Thus, we performed the Lindell and Whitney [29] marker variable procedure to assess whether common method bias was likely to affect the results. Correlations between the marker variable item and each study measure were small (ranging from –.06 to .08) and nonsignificant; thus, it is unlikely that common method bias affected the results [29,30]. The PROCESS macro (Model 6) [31] was used to test our conceptual model, including predictions involving sequential mediation [32]. The Preacher and Hayes [31] PROCESS macro for SPSS was used for several key reasons. This method uses an ordinary least squares path analysis to estimate model coefficients and assess the indirect and/or direct effects of variables in the model [32,33]. In addition, the PROCESS models use a bootstrapping procedure (n=5000), which does not rely on any assumptions about the normality of the sampling distribution, to calculate the bias-corrected 95% CIs associated with the statistical significance of the indirect effects [31,33,34]. As stated by Li and Tan [35], the Preacher and Hayes [31] PROCESS macro is “specially designed to conduct multiple mediations by including the bootstrapping function while allowing for the estimation of the indirect effect for each mediator. More importantly, this macro accounts for the effects of the control variables that are not easily implemented in structural equation modeling” (for a review of this method, see [31]). Of note, the analyses presented below were also conducted with gender as a control variable; the inclusion of gender did not impact the results presented below.

## Results

To begin, the PROCESS macro (Model 6) [31] tests the relationship between fear of COVID-19 and information receptivity. The results (*F*_1,414_=32.39; *P*<.01; *R*^2^=.07) indicate that fear of COVID-19 is positively associated with information receptivity (*β*=.48; *P*<.001). Next, the model tests whether fear of COVID-19 and information receptivity are directly associated with perceived knowledge. The results (*F_2_*_,413_=96.57; *P*<.01; *R*^2^=.32) indicate that information receptivity is directly associated with knowledge (*β*=.30; *P*<.001). However, fear of COVID-19 is not directly associated with perceived knowledge (*P*=.55). The model next tests the relationship that fear of COVID-19, information receptivity, and knowledge have with perceived self-efficacy. The results (*F*_3,412_=28.00; *P*<.001; *R*^2^=.17) show a significant relationship between knowledge and perceived self-efficacy (*β*=.55; *P*<.001). Fear of COVID-19 (*P*=.16) and information receptivity (*P*=.50) are not directly associated with perceived self-efficacy.

Next, the model tests whether fear of COVID-19, information receptivity, knowledge, and perceived self-efficacy are directly associated with prevention behaviors. The results (*F*_4,411_=11.99; *P*<.001; *R*^2^=.11) indicate that perceived self-efficacy (*β*=.44; *P*<.001), information receptivity (*β*=.25; *P*=.003), and fear of COVID-19 (*β*=.30; *P*=.02) are directly associated with prevention behaviors. However, knowledge is not directly associated with prevention behaviors (*P*=.29). Importantly, the results show support for the predicted sequential mediation (*β*=.035 [SE .014], 95% CI .012-.064), such that fear of COVID-19 is indirectly associated with prevention behaviors via information receptivity, followed by knowledge, and then perceived self-efficacy. A summary of all direct and indirect paths tested in the model is provided in Table 1.

**Table 1:** Sequential mediation results including all direct and indirect effects^a^.

Paths tested	*β* coefficient	SE	Lower limit of 95% CI	Upper limit of 95% CI
Fear of COVID-19 → information receptivity	.48	.08	.32	.65
Fear of COVID-19 → perceived knowledge	–.02	.04	–.10	.05
Information receptivity → perceived knowledge	.30	.02	.26	.34
Fear of COVID-19 → perceived self-efficacy	–.09	.06	–.21	.03
Information receptivity → perceived self-efficacy	.03	.04	–.05	.11
Perceived knowledge → perceived self-efficacy	.55	.08	.40	.70
Fear of COVID-19 → prevention behaviors	.30	.12	.06	.55
Information receptivity → prevention behaviors	.25	.08	.09	.41
Perceived knowledge → prevention behaviors	–.17	.16	–.49	.15
Perceived self-efficacy → prevention behaviors	.44	.10	.25	.64
Fear of COVID-19 → information receptivity → prevention behavior	–.12	.04	.04	.21
Fear of COVID-19 → perceived knowledge → prevention behavior	<.01	.01	–.02	.03
Fear of COVID-19 → perceived self-efficacy → prevention behavior	–.04	.04	–.12	.02
Fear of COVID-19 → information receptivity → perceived knowledge → prevention behavior	–.03	.03	–.09	.03
Fear of COVID-19 → information receptivity → perceived self-efficacy → prevention behavior	.01	.01	–.01	.03
Fear of COVID-19 → perceived knowledge → perceived self-efficacy → prevention behavior	–.01	.01	–.03	.02
Fear of COVID-19 → information receptivity → perceived knowledge → perceived self-efficacy → prevention behavior	.04	.01	.01	.06

^a^Results obtained with bootstrapping (n=5000).

Similar results were found using each of the alternative measures of prevention behaviors (alternative measure 1 [prevention behavior scale]: *F*_4,411_=90.10; *P*<.001; *R*^2^=.47; *β*=.042 [SE .012], 95% CI .021-.069; alternative measure 2 [social distancing]: *F*_4,411_=39.72; *P*<.001; *R*^2^=.28; *β*=.047 [SE .014], 95% CI .023-.077). That is, the results across three different measures of prevention behavior reveal a significant indirect relationship between fear of COVID-19 and preventive behavior.

## Discussion

### Principal Findings

Self-imposed preventive measures can drastically impact the rate of infection within a population. Public policy officials must consider the economic impact of a pandemic and be highly efficient with the limited resources that can be allocated to marketing communications [36]. While COVID-19 vaccination programs have begun, it is still of utmost importance that the public continues to take preventive measures. Indeed, simple behavioral changes taken by individuals can serve as a highly effective and low-cost method for controlling pandemics [25,37], particularly in the early stages when treatments and vaccinations are not readily available. Our findings suggest that behavioral change is largely dependent on one’s confidence in one’s own ability to execute behaviors that will reduce the likelihood of contracting or spreading the disease. Importantly, our sequential mediation results show that fear of COVID-19 leads individuals to seek out information regarding the disease, which increases their knowledge and fosters self-efficacy, a key to driving precautionary behavior. Thus, it is vitally important that government and public policy officials communicate the seriousness of the disease and the riskiness of not taking action to reduce the spread of the infection. Specifically, communications should evoke or instill fear among the public, because it is this fear that will enhance information receptivity, knowledge, self-efficacy, and ultimately changes in behavior.

Of note, our results show that fear of COVID-19 itself is not directly associated with preventive behavior. Instead, fear of COVID-19 was only associated with preventive action when individuals sought out knowledge of appropriate preventive measures and felt confident in their ability to take such actions. As stated by Brug et al [38], “risk perceptions as well as efficacy beliefs in the early stages of a possible pandemic are dependent on communications with and between the members of the groups at risk. Risk communication messages that are not comprehended by the public at risk, or communication of conflicting risk messages will result in lack of precautionary actions.” The widespread confusion over the efficacy of wearing masks during the current pandemic is one such example.

A system needs to be in place to disseminate accurate information about the virus and appropriate precautionary measures that individuals should take. A system also needs to exist to deter, counteract, and quickly terminate any misinformation that may be spread through news outlets and, more likely, through social media. It is crucial that the information offered is not only accurate but also trustworthy. Thus, consistency is key, and an integrated marketing communications approach would be helpful. Indeed, research has shown that repetitive mentions of preventive actions can serve to reduce fear surrounding a disease, and ultimately increase preventive behaviors, whereas the opposite is true when individuals question the trustworthiness of the information [39].

The overarching objective of this study was to create and test a model for predicting COVID-19 preventive behaviors that addresses the shortcomings of the HBM, and to use this information to formulate more effective health communications to increase the adoption of behaviors that slow the spread of COVID-19. The HBM has a long history of use and empirical support in predicting and explaining a range of health-related behaviors [19]. However, an improved model is needed, given criticisms that note the explanatory abilities of the HBM need to be improved. The resulting sequential mediation model presented herein answers the call for a better definition of the relationships between the antecedents of health-seeking behavior. The present model includes the measure of fear of COVID-19, which adds a validated scale that addresses the COVID-19 pandemic specifically. A criticism of the commonly used scales of the HBM is that they have not been adequately validated [5]. A valid scale to measure the perceived threat and fear of COVID-19 is essential given the important role perceived susceptibility and fear of a disease play in getting individuals to act to lessen their risk of contracting a disease or avoiding other negative health outcomes.

The use of three separate measures of COVID-19 health-seeking behaviors, including items to measure social distancing, is also an important contribution of this study. Items of the Manika and Golden [16] prevention behavior scale were answered on a 7-point Likert scale, while the Masuri et al [17] scale was a list of questions about health-related behaviors that were answered with a “yes” or “no” response. The 2-item social distancing scale added two behaviors (social distancing and quarantining) that were not part of the other two scales but are reflective of the primary thrust of current preventive message campaigns to social distance to protect oneself and to avoid spreading COVID-19.

Data generated from the current survey provide clear directions on how to best encourage COVID-19 preventive behaviors. The present model begins with fear of COVID-19. This is consistent with the HBM model, which states that fear of the disease or another health problem must be present and that individuals must perceive that they are susceptible to the disease or health problem [8]. A good example of the importance of fear of and susceptibility to COVID-19 is evident in young adults, especially college students. Given the general tendency of the young to feel a certain sense of invulnerability to health risks, it has been important to inform them that, although they might not be at high risk for severe disease if they were to contract COVID-19, they could spread the disease to their parents, grandparents, and other older adults. According to Orji et al [21], people of all ages tend to underestimate their likelihood of contracting diseases or experiencing other health problems. Thus, it is important that COVID-19 marketing communications stress the high transmissibility of the virus (susceptibility), as well as the potential severity of the disease (fear).

As suggested by Manika and Golden et al [16], these “fear appeal” messages must be consistent across all media. Conveying consistent and clear messages of the dangers of COVID-19 is made difficult by social media. Research has shown that people are exposed to considerable amounts of misinformation online, which can undermine the effectiveness of public health campaigns [40,41]. Social media health campaigns are critical to informing the public about proper health behaviors while also dispelling any misinformation that may be being spread online.

This study found that fear of COVID-19 was positively associated with information receptivity. Information receptivity measures how actively an individual searches for COVID-19–related information. Manika and Golden [16] found that people who were more receptive to H1N1 information were also more likely to engage in H1N1 prevention behaviors. The authors concluded that being receptive to information signals an “individual’s readiness to act, thus it is more likely that the individual will take appropriate prevention measures.” The importance of information receptivity underlines the necessity of having consistent and clear messages—delivered across all media—regarding what types of behaviors are recommended to increase compliance.

In this model, greater information receptivity, not surprisingly, is associated with higher levels of perceived knowledge. The model used perceived knowledge, as opposed to stored (or actual) knowledge, given that research has found that it is one’s perceived knowledge (not stored) that leads to requested health-related behaviors [16]. This study also found that perceived knowledge is positively associated with self-efficacy. The more a person believes he or she is equipped with the needed knowledge regarding COVID-19, the more confident they will be that they can perform the behaviors needed to protect themselves from contracting the virus. Health messages must be clear about what types of behaviors are needed and the ease of performing those behaviors.

The public needs to be convinced that behaviors such as washing their hands, wearing masks, social distancing, and self-quarantining will lead to a valued outcome (eg, avoiding contracting COVID-19, reopening the economy). Self-efficacy must be at the forefront of any media campaign. This is particularly true given that self-efficacy is the final variable in our sequential mediation model driving COVID-19 preventive behaviors.

This study used three separate measures of COVID-19 preventive behaviors and found that the 4-item measure developed by Manika and Golden [16] worked the best. The *R*^2^ of .47 suggests that the model does a good job of explaining variation in COVID-19 prevention behavior, but also that a significant percentage of such behavior remains unexplained. It could be, as exemplified by Orji et al [21], that new variables need to be added to the present model. A higher *R*^2^ value may also be achieved by maintaining the current variables of the present model while also including variables already designated in the HBM but not used in this study. Cues to action such as experiencing possible symptoms of COVID-19, conversations among friends and colleagues, and the amount and type of media exposure would all likely increase the explanatory capability of the model. Additionally, a broader array of sociodemographic measures would also increase the *R*^2^ value. Although not found in this study, sex differences have previously been found, with females more anxious about the disease and more likely to perform preventive health behaviors. Age, socioeconomic status, and ethnicity have been identified as possible factors that impact health-related behaviors and should be incorporated into future models.

### Limitations and Future Research Directions

Although this survey is the first to apply a sequential mediation model to better understand COVID-19 preventive behaviors, it is not without limitations. First, the sample was from only one segment of the population, albeit an important one. Future research should sample older adults and younger adults, as well as people from specific ethnic populations. In the United States, the African American and Latino populations have been impacted more severely than the White population. A survey of these segments of the population with the present model might provide insights into what encourages or discourages the needed preventive behaviors across ethnic/racial groups. In addition, future research should consider how individuals’ backgrounds, medical histories, and other individual difference variables, including those related to public health knowledge, may impact the results found as well as the general performance of the HBM.

A second limitation is the study’s correlational nature. Future causal and/or longitudinal research is needed that can test the links in the present model and provide more reliable insights into the direction of the causal flow. A third possible limitation and fodder for future research is the further expansion of the present model. As called for by Jones et al [20], a deeper investigation of cues to action could improve explanatory capacity. Internal cues such as perceived COVID-19 symptoms and external cues such as media exposure (both amount and type) might be better addressed separately. As Jones et al [20] suggest, it might also prove beneficial to investigate manipulated cues to action like public service campaigns, media messages, and interventions, as well as more organic cues to action like illness in the family, discussions among friends and family, news stories, and high profile people contracting the disease.

### Conclusion

Drawing upon earlier attempts to explain health-seeking behavior, this study created and tested a sequential mediation model to explain COVID-19 preventive behaviors. The new model’s *R*^2^ was .47, which is considerably higher than the average *R*^2^ of approximately .20 across numerous studies using the HBM [21]. The study also identified a better-defined model of the relationships between its predictor variables. Both results were in response to calls to improve the purported shortcomings of the HBM in predicting health-related behavior. Finally, this study’s results provide clear directions for creating effective strategies and messages to encourage the behaviors needed to slow, if not eradicate, the most serious pandemic of the past 100 years.

## Abbreviations

EVT: Expectancy-Value Theory
HBM: Health Belief Model
MERS: Middle East respiratory syndrome
SARS: severe acute respiratory syndrome
SCT: Social Cognitive Theory
